# miR-301a mediated immune evasion by Japanese encephalitis virus

**DOI:** 10.18632/oncotarget.21674

**Published:** 2017-10-09

**Authors:** Bibhabasu Hazra, Surajit Chakraborty, Anirban Basu

**Affiliations:** Bibhabasu Hazra: National Brain Research Centre, Manesar, Haryana, India

**Keywords:** Japanese encephalitis virus, neuron, innate immunity, microRNA, IRF1

Japanese encephalitis virus (JEV) is the most leading cause of acute viral encephalitis in Asia-Pacific region. It principally targets central nervous system (CNS) and is clinically manifested by headache, vomiting, impaired consciousness, and often seizures. Approximately, 20-30% of patients die annually and nearly half of the survivors were left with permanent neurological sequelae [1]. Although multiple vaccines are available, JEV cases are continuously being reported every year. There are no effective antiviral therapies and treatment is mainly supportive to relieve the clinical symptoms.

Amongst the important reasons of failure to control viral infection is their ability to adopt modified strategy to escape host immune response. Antiviral innate immunity includes induced expression of type I interferons (IFNs) is the most vital and primary part of host immune system to deliver first line of defence against virus. Though it is a spontaneous response to invading virus, expression of innate immune molecules is reported to be influenced by several gene regulatory mechanisms including post-transcriptional regulation by microRNAs (miRNAs). miRNAs are small non-coding RNA molecules that have been identified as a key regulator in almost every cellular processes. In case of viral infections, they serve as a fine-tuner in regulation of both cellular and viral gene expression as evidenced from some recent studies [2].

Very recently, our work demonstrated that host miR-301a induced in early JEV infection suppresses antiviral IFN-β response in neurons [3]. Inhibition of miR-301a in JEV infection resulted in significant restoration of IFN-β production and restricts viral replication. Furthermore, IFN-stimulated genes (ISGs) that mediate the antiviral activity of IFN-β were also induced by miR-301a inhibition.

Deciphering the role of elevated miR-301a expression in virus induced host response seemed crucial for gaining insight into probable ways of managing JEV infection. We identified IFN regulatory factor 1 (IRF1) and suppressor of cytokine signalling 5 (SOCS5) as prospective targets of miR-301a which potentially regulate host antiviral IFN response. Increased expression of miR-301a in neuron resulted in significant reduction of both these proteins and conversely miR-301a knockdown restored their expression in JEV infection. Evidences demonstrate IRF1 as a potent inhibitor of flavivirus infections and regulate both innate and adaptive arms of immune response [4]. Binding of IRF1 to the regulatory region of IFN-β gene induces expression of ISGs followed by the initiation of antiviral response [5]. Knock down or over-expression of IRF1 in miR-301a inhibited condition is characterized by reduced or enhanced expression of IFN-β in JEV infected neuronal cells respectively.

The expression level of another miR301a target SOCS5 was observed to undergo reduction upon JEV infection. Usually viral infections are reported to elevate SOCS gene expression, which differed from our results. However, a report showed that augmentation of SOCS5 empowers the innate immunity further in murine model of septic peritonitis and improves survival [6]. Likely, we noticed that over-expression or inhibition of SOCS5 in JEV-infected neuron respectively enhanced or decreased IFN-β expression in miR-301a inhibited condition. But the exact role of SOCS5 in IFN-β regulation was unknown. Based on some previous studies [7], we observed that decrease in SOCS5 abundance in JEV infected neurons induces epidermal growth factor receptor (EGFR) activation and thereby inhibiting IRF1 expression. Furthermore, repression or elevation of SOCS5 reduced or induced IRF1 expression in JEV infected neurons respectively. Improved response of IRF1 mediated IFN-β in cells treated with EGFR inhibitor AG 1478 also underscores the importance of SOCS5 involvement in IRF1 mediated IFN-β expression upon JEV infection. Since one miRNA can regulate a thousand of genes, targets other than IRF1 and SOCS5 may have significant roles in miR-301a regulation of JEV infection.

Restoring miR-301a aberrant expression in JEV infection to its normal pattern would help in providing an effective strategy against the same, thus further warranting investigation of factors regulating miR-301a expression in JEV infection. We found an authentic NF-κB binding site in miR-301a promoter and degree of miR-301a expression was directly proportional to the extent of NF-κB activation in JEV infected neuronal cells. Consistently, restricting NF-κB translocation in JEV infected neuron resulted in reduced expression of miR-301a and enhanced abundance of IRF1 and SOCS5, which signifies that JEV induced NF-κB promotes miR-301a expression in neuronal cells.

We also investigated the regulatory role of miR-301a in JEV infected mouse model. We treated JEV infected mice with miR-301a Vivo-Morpholino (miR-301a-VM) for effective silencing of miR-301a. Consistent with *in vitro* results, inhibition of miR-301a rescued IRF1 and SOCS5 expression followed by induction of interferon response and thus efficiently reducing viral load in JEV infected mouse. Moreover, miR-301a-VM treatment ameliorated signs including paralysis, loss of body weight in addition to increased survivability of mouse models. Although JEV initially infects peripheral organs including liver, kidney, heart, lung followed by CNS, we did not notice any regulatory effect of miR-301a on IFN production in those organs.

**Figure 1:**
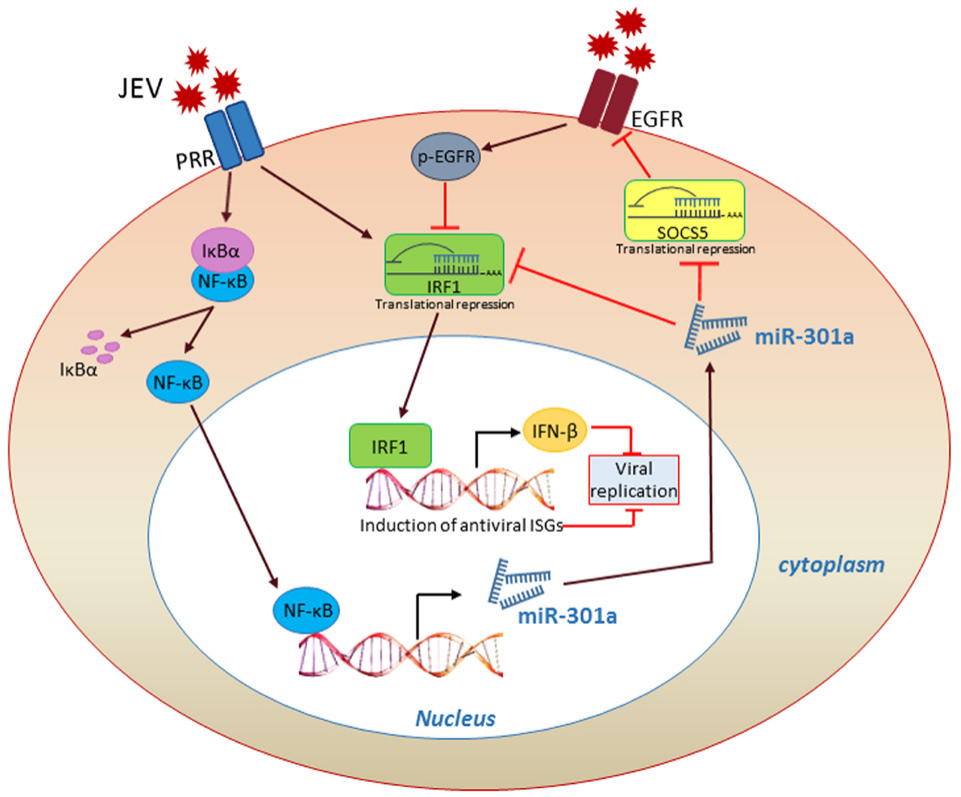
Activated NF-κB in JEV infection induces miR-301a, which weakens IFN-β response through suppression of IRF1 and SOCS5.

In conclusion, we have characterized a miRNA mediated virus-host interplay where JEV infection induces NF-κB activation-dependent expression of miR-301a which help virus to evade immune surveillance by suppressing IRF1 and SOCS5 and thus weakening the IFN-β response (Figure 1). Neutralization of JEV induced miR-301a hinders this signalling pathway, thereby rescues host IFN response and improves survival in JEV infected mice. In future, using miR-301a as a potential therapeutic target in JEV infection would be a vital outcome of our study. Our findings shed light on the role of host miRNA in virus induced IFN response and also help us to figure out the molecular mechanism of viral pathogenesis.
